# Fluoroscopic Stenting as a Bridge to Surgery *versus* Emergency Management for Malignant Obstruction of the Colon

**DOI:** 10.1155/2020/4650780

**Published:** 2020-06-01

**Authors:** Fan Xue, Feng Lin, Jun Zhou, Ning Feng, You-Gang Cui, Xu Zhang, Yu-Peng Yi, Wen-Zhi Liu

**Affiliations:** ^1^Department of Gastrointestinal Surgery, Affiliated Zhongshan Hospital of Dalian University, Dalian 116001, Liaoning Province, China; ^2^Department of Intervention, Affiliated Zhongshan Hospital of Dalian University, Dalian 116001, Liaoning Province, China

## Abstract

**Aim:**

To investigate the feasibility of a SEMS (self-expandable metallic stent) as a bridge to surgery for malignant colonic obstruction.

**Methods:**

We retrospectively reviewed 83 patients that were in accordance with inclusion criteria; of these, 33 patients that underwent fluoroscopy-guided SEMS placement followed by elective curative resection were classified as a SEMS group and 50 patients, who received emergency surgery (ES), were classified as an ES group. The clinicopathological characteristics, surgery-related parameters, complications, and three-year survival rate were compared between the two groups.

**Results:**

No significant differences between the two groups were observed in any of the clinicopathologic characteristics except for higher preoperative absolute neutrophil count in the ES group (*P* < 0.001). Compared to the ES group, the SEMS group has significantly more cases, which featured a laparoscopic approach (72.7% vs. 14.0%, *P* < 0.001), lower overall stoma rate (0% vs. 34.0%, *P* < 0.001), and lower overall postoperative morbidity (27.3% vs. 56.0%, *P*=0.010). The oncological outcomes did not differ significantly between the two groups in terms of three-year overall survival (*P*=0.125). The technical and clinical success rates of stent placement were 91.7% and 100%, respectively.

**Conclusion:**

Patients treated with the stent-surgery approach had significant short-term superiorities and similar long-term outcomes, compared to patients who had emergency surgery alone. The SEMS is, therefore, safe and feasible as a bridge to surgery for malignant colonic obstruction.

## 1. Introduction

Colorectal cancer is the third and second most common global cancer in males and females, respectively, and is showing increasing levels of morbidity in China [1, 2]. Obstruction is a serious complication of advanced colon cancer and occurs in 8–13% of patients [2, 3]. The traditional intervention for this condition is emergency surgery; however, emergency surgery is accompanied by high postoperative morbidity (45–50%) and mortality (15–20%) accounting for uncorrected pathophysiological changes and aggravation of underlying diseases [4].

The self-expandable metallic stent (SEMS) has been applied in clinical practice since the 1990s, which is an alternative form of emergency treatment for malignant colon obstruction. The SEMS is considered to function as a bridge to elective resection and allow the preparation for surgery in a much more efficient manner. The results of several nonrandomized studies have demonstrated that the placement of SEMS, combined with elective resection, can significantly improve short-term outcomes, including a lower postoperative complications rate and a shorter hospital stay [5–9], and better long-term outcomes [10]. However, other researchers have reported contradictory results. Some authors considered that the complications associated with SEMS placement would increase morbidity, mortality, and surgical difficulty [11–13], while others reported that SEMS placement was associated with inferior long-term oncological outcomes [14, 15]. Consequentially, the combination of the SEMS and elective resection is still a controversial management strategy for malignant colon obstruction.

The aim of our study was to investigate the feasibility of a SEMS as a bridge to surgery for malignant colonic obstruction by comparing the short- and long-term outcomes of the patients treated with the SEMS combined with elective surgery to those with emergency surgery only.

## 2. Methods

### 2.1. Patients

The design of our study was approved by the Institutional Review Board (no. 2017209). We retrospectively reviewed patients who were admitted in our hospital between 2010 and 2017. Patients who met the following inclusion criteria were included in the study: pathologically diagnosed with colon cancer, either before or after surgery; accompanied by acute obstruction which was determined by clinical manifestations, physical examinations, and abdominal imaging examinations; performing SEMS placement or emergency surgery with curative intent; resectable synchronous liver metastasis and local advanced cancer were indications for surgery with curative intent. Patients who were diagnosed with obstruction combined with perforation were excluded from the study.

**Figure 1:**
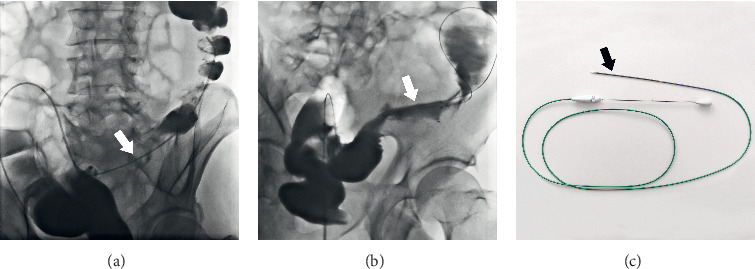
(a) Filling defect showed the location and length of lesion (arrow). (b) A stent (arrow) was placed and obstruction was relieved after the procedure. (c) A constricted SEMS (arrow) on the conveyer.

Either SEMS placement or emergency surgery was performed if symptoms persisted or worsened after attempting conservative treatments within 72 h. The choice between these two management styles was made by the attending surgeon and by patient preference. All the SEMSs in our study were inserted by three experienced interventional radiologists under fluoroscopic guidance of a digital subtraction angiography machine. A catheter was placed through the anus to the distal point of the lesion, and a contrast medium was injected to visualize the lumen of the colon. We then inserted a standard or hydrophilic guidewire through the lesion. We estimated the length of stenosis and delivered a suitable uncovered SEMS using a superstiff guidewire through the lesion. The SEMS was then released after confirming that the two ends of the stent extended 2-3 cm beyond the margins of the stricture (Figure 1). If the procedures described above were completed, the procedures were defined as a technical success. Subsequently, clinical success was defined as when the symptoms of obstruction were alleviated completely and when the patient was successfully able to take oral laxatives for bowel preparation before surgery. If either technical failure or clinical failure occurred, then the patient would undergo emergency surgery instead. After complete remission of colonic obstruction, we would carry out preoperative assessments and preparations. The interval between SEMS placement and elective surgery was decided by the attending surgeon according to the patient's individual condition. Then, an elective surgery would be performed by two dedicated colorectal surgical teams, including resection of the colon segment where the lesion was located with primary anastomosis, routine mesocolic lymphadenectomy, and curative metastasectomy if metastasis presented. Preoperative imaging examinations and intraoperative exploration were performed to screen multisite primary cancers.

The type of emergency surgery carried out was determined by three experienced general surgeons depending on intraoperative findings and the individual condition of the patient following colonic lavage during surgery. Generally, one-stage radical resection, or Hartmann's procedure, was attempted.

### 2.2. Clinical and Laboratory Data

The baseline characteristics of the patients in the two groups were collected from the hospital database, including age, sex, previous/preexisting diseases, body mass index (BMI), American Society of Anesthesiologists (ASA) class, oncological information, and laboratory examinations prior to surgery. The condition of previous/preexisting diseases was externalized as Charlson's weighted index of comorbidities (WIC) and oncological information, including tumor location, pathological stage, differentiation degree, and perineural/vascular invasion. Surgical characteristics included operative approach, resection margin, stoma formation, surgery duration, and intraoperative blood loss. Postoperative complications were classified into five levels of severity according to the Clavien–Dindo grading system. As paregoric or total parenteral nutrition was routine postoperative prescription for patients with colonic cancer, they were not counted as postoperative complications in this study. The SEMS-related characteristics were also collated, including technical success, clinical success, interval between stent and surgery, and stent-related complications. All patients were followed up periodically until death or March 2020, in adherence to the follow-up strategy described by the National Comprehensive Cancer Network (NCCN) Guidelines. Three-year overall survival, as long-term outcomes, was then compared between the two groups.

### 2.3. Primary and Secondary Outcomes

Primary outcomes of our study were short-term outcomes, including postoperative length of hospital stay and overall postoperative complication, as well as long-term outcomes and the three-year overall survival. Secondary outcomes were technical success, clinical success, and surgical characteristics.

### 2.4. Statistical Analysis

All statistical analyses in this study were conducted using IBM SPSS version 22.0 (IBM Co., Armonk, NY, USA). Categorical variables were reported as frequencies and percentages. The chi-square test, or Fisher's exact test, was used for comparisons between groups. Continuous variables were described using means and standard deviations if normally distributed and compared using an independent *t*-test, otherwise medians with interquartile ranges were applied, and comparison was made using the Mann–Whitney *U* test. Analysis of three-year overall survival was conducted by Kaplan–Meier statistics, and comparison of the two groups was performed by a log-rank test. *P* < 0.05 was considered to be statistically significant.

## 3. Results

### 3.1. Patient Characteristics

A total of 210 patients diagnosed with malignant colon obstruction treated with SEMS placement (the SEMS group, *n* = 132) or emergency surgery (the ES group, *n* = 78) were identified between 2010 and 2017. Of these, 96 patients in the SEMS group and 27 patients in the ES group were excluded as they received palliative treatment, and 4 patients were excluded as they were diagnosed with perforation. Three patients were initially referred for stenting; however, they were converted to emergency surgery due to technical failure (8.3%). The three failure cases all involved a failure to pass a guidewire across the lesion; in all cases, lesions were located in the right-sided colon. Only one case of the SEMS-related complication occurred; this involved migration of the stent. It was interesting; however, we realized this fact as a intraoperative finding. Finally, 33 and 50 patients were classified to the SEMS group and the ES group, respectively. All study participants, or their legal guardian, provided informed written consent prior to study enrollment. The technical and clinical success rates were 91.7% and 100%, respectively, and the rate of stent-related morbidity was 3%.

As summarized in Table 1, there were no significant differences between the two groups in any of the baseline characteristics, except for preoperative absolute neutrophil count (*P* < 0.001). In both groups, the tumors were mostly located in the sigmoid colon, but the distribution of tumors in the ascending colon was relatively higher in the emergency surgery group. Liver metastases were found by preoperative examinations in five patients (three in the SEMS group and two in the ES group), and all were resected during surgery.

### 3.2. Comparison of Clinical Outcomes

The median interval from stent placement to elective surgery was 14 days (range, 7 to 34 days). The types of surgeries performed are summarized in Table 2. In terms of operative approach, there were significantly more laparoscopic surgeries in the SEMS group (72.75% vs. 14.0%, *P* < 0.001). Temporary or permanent stoma were fitted in 17 patients, including 14 Hartmann's procedures and three ileostomies in the ES group, but no stoma was fitted in any of the SEMS patients (*P* < 0.001). *R*_0_ resection was attempted in all patients; however, the resection margins of seven patients were confirmed to be positive by microscopic examination after surgery (6.1% *vs.* 10.0%, *P*=0.819). The mean surgical length in the SEMS group was shorter than that of the ES group, but there was no statistically significant difference between the two groups in this respect (190.4 min vs. 209.5 min, *P*=0.182). Furthermore, there were significant differences in terms of intraoperative blood loss and postoperative hospital stay when compared between the two groups (210.6 mL *vs.* 310.2 mL and 13.3 d vs. 18.5 d, *P*=0.036 and *P*=0.020, respectively). In the SEMS group, four patients were admitted to the intensive care unit (ICU) after surgery and four postoperative complications were observed; of these, one case was due to incision rupture and two because of anastomotic leakage and gastroparesis, respectively, and one case of intra-abdominal hemorrhage was cured by angiointerventional embolization. In the ES group, 18 patients were admitted to the ICU due to single or multiple organ dysfunction; of these, three patients died from septic shock and one patient died due to anastomotic leakage. Five cases of incision infection, two cases of bowel obstruction, two cases of pneumonia, and one case of myocardial infarction were observed within 30 days of surgery. Adding one case involving stent-related complications, the overall postoperative morbidity was 27.3% (9/33) in the SEMS group and 56.0% (28/50) in the ES group (*P*=0.010). Results relating to grade I–V on the Clavien–Dindo classification of surgical complications are summarized in Table 3. There was no significant difference between the two groups in terms of grade I (*P*=0.443), II (*P*=1.000), or III (*P*=1.000); however, there were significantly more patients in grade IV in the ES group than in the SEMS group (*P*=0.016). While four grade V complication cases were observed in the ES group (compared to none in the SEMS group), there was no statistical difference between the two groups in this respect (*P*=0.254).

Since three patients initially referred for stenting were converted to the ES group due to technical failure, which could bias the results for short-term outcomes, we performed intention-to-treat (ITT) analysis as a sensitive analysis. The ITT showed that the SEMS group had a higher odds of minimally invasive surgery (*P* < 0.001), higher primary anastomosis rate (*P* < 0.001), and lower overall postoperative morbidity (*P*=0.007), and no significant differences existed in the length of surgery (*P*=0.290), intraoperative blood loss (*P*=0.061), and postoperative hospital stay (*P*=0.523). Similar results were also reached relating to grade I–V on the Clavien–Dindo classification, which were grade I (*P*=0.346), II (*P*=0.930), III (*P*=0.999), IV (*P*=0.023), and V (*P*=0.808).

### 3.3. Comparison of Long-Term Survival

**Figure 2:**
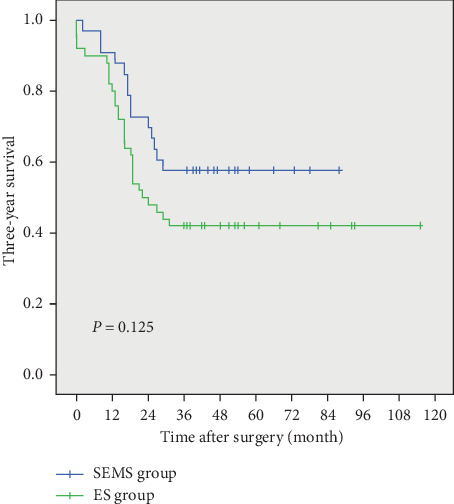
Kaplan–Meier analysis. Between the SEMS group (blue line) and ES group (green line), there were no significant differences in three-year overall survival (*P*=0.125).

The mean follow-up time was 25.0 ± 17.1 months for the SEMS group, which was not significantly different from that of the ES group (25.2 ± 23.1 months, *P*=0.969). Based on per-protocol (PP) analysis, the 3-year survival rate of the SEMS group was not significantly different from the ES group (57.6% vs. 42.0%, *P*=0.165), with a median survival time of 37.0 (18.0–52.0) months in the SEMS group compared to 23.0 (13.8–44.3) months in the ES group (*P*=0.313) (Figure 2). The ITT analysis showed a similar result that there was no significant difference in 3-year survival rate between the SEMS group and ES group (55.6% vs. 42.6%, *P*=0.234) with a median survival time of 37.0 (17.3–50.0) and 24.0 (14.0–48.0) months, respectively (*P*=0.582).

## 4. Discussion

PP and ITT analyses in our study yield similar results that the SEMS, as a bridge to surgery, had a higher likelihood of minimally invasive surgery and a higher primary anastomosis rate and exhibited a favorable short-term outcome compared to emergency surgery due to a lower overall postoperative morbidity. In terms of long-term outcomes, the SEMS plus elective surgery did not lead to a significant improvement in 3-year survival compared with emergency surgery alone, based on PP and ITT analyses. As summarized in Table 4, our results were similar to those described in most of the previous systematic reviews and meta-analyses [4, 16–29]. However, some controversy remains with regards to the short- and long-term impact of the SEMS as a bridge to surgery.

There has been some concern with regards to short-term outcomes as to whether the SEMS can reduce overall postoperative mortality and morbidity. Although overall postoperative mortality did not differ significantly between the two groups (0% vs. 8%, *P*=0.254), emergency surgery tended to increase the mortality since four death cases were observed in the ES group (compared to none in the SEMS group). The mortality after emergency surgery for obstructive colorectal cancer was reported as 15–20% [4], while postoperative mortality after colorectal cancer surgery was 5.8% in a cross-sectional population-based study [30]. The higher mortality in the former study could mean that emergency surgery was a risk factor for postoperative mortality, which needed to be confirmed in future studies. The results of our study showed that overall postoperative morbidity was significantly lower in the SEMS group (27.3% *vs.* 56.0%, *P*=0.010). There were some reasons we believed, which could explain the lower overall postoperative morbidity in the SEMS group. Firstly, patients in the SEMS group had a relatively lower preoperative neutrophil count (4.4 (3.2–5.3) vs. 7.6 (5.5–9.8), *P* < 0.001), which could indicate a less severe systemic inflammatory response. Neutrophil count was proved to be positively related to the postoperative complication rate of patients with colorectal cancer [31]. Secondly, the SEMS, as a bridge to surgery, made time for more comprehensive preoperative assessments and more sufficient preoperative preparations, which could lead to a better short-term outcome. Thirdly, fewer stent-related complications in our study also contributed to the outcome; otherwise, overall postoperative morbidity would be raised to some extent. Previous meta-analyses, relied solely on data from randomized trials, showed similar results that the SEMS group had significantly lower overall postoperative morbidity compared to the ES group, and there was no significant difference in terms of postoperative mortality [20, 21, 24, 27]. However, a recent multicenter randomized controlled trial demonstrated that there were no statistical differences regarding to the postoperative complication rate within 60 days when compared between the SEMS and ES (51.8% vs. 57.6%, *P*=0.529) [15]; similar results were observed in two other randomized trials [13, 32]. Moreover, the results of Min et al. [11] showed that the SEMS increased postoperative morbidity rather than causing a reduction (8.3% in a subtotal colectomy group vs. 31.4% in the SEMS group, *P*=0.025) and no statistical difference in mortality between the two groups (0% vs. 2.9%, *P*=1.000). Elective surgery followed SEMS placement was associated with fewer postoperative morbidities than emergency surgery in above studies; therefore, widely different morbidities of stent-related complications result in the divergences among the research studies.

The incidence of general complications related to stent placement, such as perforation, migration, and reobstruction, was reported as 0–14.1%, 0.9–21.9%, and 0.5–40%, respectively [12]. Incidence of stent-related perforation is even higher in some studies, and SEMS placement is no longer recommended by some experts in the setting of obstructive colorectal cancer [3]. However, we did not observe incidence of clinical perforation in our study; the reason for this, in our opinion, is the approach for SEMS placement. Approaches of colonic stent placement include fluoroscopy alone, colonoscopy alone, and a combination of fluoroscopy and colonoscopy. The colonoscopy has greater accessibility to the lesion and leads the guidewire into stenosis more easily, while fluoroscopy is useful for visualizing the length of an obstruction and detection of a perforation. Few studies have directly compared complication rate between endoscopic and radiologic SEMS placement. Kim et al. [33] reported that complication rate did not differ statistically between two methods (32.4% vs. 15.4%, *P*=0.303). However, either colonoscopy alone or a combined method, in our opinion, is unsafe for the patients with obstruction. On one hand, since satisfactory bowel preparation is almost impossible for a patient with obstruction, the access to lesion under colonoscopy is difficult and dangerous due to an unclear view. On the other hand, the trend of guidewire cannot be visualized under colonoscopy when passing through the stenosis, which may increase the risk of perforation. Instead, fluoroscopy approach avoids the two problems mentioned above and be readily capable of defining whether perforation is present. In the study reported by Kim et al. [33], the result showed that radiological methods appear to avoid the occurrence of perforation, although there was no statistically difference compared to endoscopy. Moreover, other factors could also have an impact on the incidence of stent-related complications, including the experience of the surgical team, stent type, tumor location, neoadjuvant chemotherapy, and time interval to surgery. For the reasons outlined above, notable divergences in the stent-related morbidities have been described in the present literature.

The SEMS, as bridge to surgery for obstructive right colon cancer (ORCC), is also controversial approach. There were nine patients with ORCC in our study that were initially referred for stenting on account of their poor condition or willingness, while three of them were converted to emergency surgery due to technical failure. In our study, there was no significant difference in terms of short- and long-term outcomes between patients with ORCC in two groups based on PP and ITT analyses, which was similar to the results of a recent multicenter retrospective study [34]. However, emergency surgery is still the preferred option for most surgeons, which may be related to several anatomical reasons, including relatively easier to mobilize hepatic flexure compared to splenic flexure, convenient to anastomose intestines due to the great mobility of small bowel, and safe to perform primary anastomosis benefitting from the optimal blood supply. Adding that SEMS placement is considered to be technically difficult for ORCC, right colectomy with primary anastomosis is recommended by guidelines [3, 35]. Nevertheless, the optimal management of ORCC is still pendent until more convincing evident is raised.

The long-term outcome of the SEMS has always been the source of much scrutiny. Recent meta-analyses have reported no differences in the long-term oncological outcomes when comparing SEMS and ES patients [23, 29]. Our current study showed a similar result in that the oncological outcomes did not significantly differ between the two groups in terms of the three-year overall survival rate (57.6% vs. 42.0%, *P*=0.125). However, the oncological safety of SEMS placement has always been a topic of some debate, and several studies have reported the negative impact of the SEMS upon oncological outcomes. In previous studies, Sabbagh et al. reported that overall survival and 5-year overall survival were significantly lower in the SEMS group compared with the ES group [36], and three randomized controlled trials have reported a tendency of decreasing in disease-free survival [37–39]. Especially of these, Alcántara et al. [37] reported a significant high rate of recurrence in the SEMS group (53.3%) compared to the ES group (15.4%). A similar result was concluded by Gorissen et al. that the SEMS group was associated with a higher local recurrence rate than the ES group (32% *vs.* 8%, *P*=0.038), and they hypothesized that the cause was tumor dissemination induced by stent-related subclinical perforation [40]. The mechanical compression on a tumor generated by the SEMS is believed to not only increase the risk of perforation [41] but also elevate the level of circulating tumor cells and induce early distant metastases [14]. Considering these divergent results, the long-term oncological outcomes of the SEMS as a bridge to surgery for malignant colonic obstruction is still controversial. Further higher quality studies, involving a larger sample size of patients and a design for long-term follow-up, are needed in the future, and basic experimental research is needed to provide more insight into the effect of SEMS placement on the biological behavior of tumors.

There are some limitations to this study which need to be considered when interpreting our findings. Firstly, the patients were assigned to different management styles by attending surgeon or patient preference rather than randomization. This could result in selection bias because surgeons tended to choose a stent for the patients with poor condition, while patients preferred the emergency surgery due to economic considerations. Secondly, the data from this study were limited to what were recorded in the medical records in our institution. It is possible that patients received treatment (e.g., adjuvant chemotherapy) and follow-up care from other hospitals that we had no access to these data. In addition, the length of the follow-up period after surgical procedure might not be long enough to observe long-term survival outcomes for both groups. All patients were from a single health center in a metropolitan area. Therefore, our findings may not be generalized to other patient populations. A multicenter, longer, larger-scale randomized controlled study is needed to comprehensively compare the efficacy and short- and long-term outcomes between SEMS and ES procedures.

In conclusion, the results of our study showed that the SEMS, as a bridge to surgery for malignant colonic obstruction, was safe and feasible. Compared to emergency surgery, patients treated with stent-surgery exhibited significant short-term improvement and comparable long-term outcomes. We suggest that the SEMS, combined with elective surgery, is a promising alternative for patients with resectable malignant colon obstruction.

## Figures and Tables

**Table 1:** Comparison of variables between the SEMS group and ES group.

Variables	SEMS group (*n* = 33)	ES group (*n* = 50)	*P* value^‡^
Age (years)^†^	73 (62–77)	70 (60–80)	0.867^¶^
Gender			0.343
Male	25 (75.8)	33 (66.0)	
Female	8 (24.2)	17 (34.0)	
WIC^*∗*^	4.1 (2.4)	3.7 (2.4)	0.433^§^
BMI^*∗*^	22.4 (2.8)	22.3 (3.6)	0.946^§^
ASA (%)			0.612
I	1 (3.0)	5 (10.0)	
II	22 (66.7)	27 (54.0)	
III	9 (27.3)	15 (30.0)	
IV	1 (3.0)	3 (6.0)	
Tumor location			0.194
Ascending colon	4 (12.1)	16 (32.0)	
Transverse colon	2 (6.1)	2 (4.0)	
Descending colon	10 (30.3)	13 (26.0)	
Sigmoid colon	17 (51.5)	19 (38.0)	
Pathological stage			0.627
I	2 (6.1)	2 (4.0)	
II	15 (45.4)	21 (42.0)	
III	13 (39.4)	25 (50.0)	
IV	3 (9.1)	2 (4.0)	
Differentiation degree			0.309
Well-moderate	31 (93.9)	42 (84.0)	
Poor-undifferentiation	2 (6.1)	8 (16.0)	
Perineural invasion	5 (15.2)	8 (16.0)	0.917
Vascular invasion	8 (24.2)	13 (26.0)	0.857
Absolute neutrophil count (10^9^/L)^†^	4.4 (3.2–5.3)	7.6 (5.5–9.8)	<0.001^¶^
HGB (g/L)^†^	120.0 (107.5–134.5)	131.5 (104.3–147.5)	0.161^¶^
ALB (g/L)^†^	36.5 (34.3–39.2)	38.8 (34.1–43.3)	0.163^¶^
CEA (ng/ml)^*∗*^	17.2 (23.8)	28.1 (67.9)	0.376^§^
Time of stent placement (mins)	88.8 (44.3)	—	—

Values are presented as number (%). ^*∗*^Mean (s.d.) and ^†^median (i.q.r.). ^‡^ Chi-square or Fisher's exact test, except ^§^ independent *t*-test and ^¶^ Mann–Whitney *U* test. SEMS, self-expanding metal stent; ES, emergency surgery; WIC, Charlson's weighted index comorbidities.

**Table 2:** Types of surgeries performed.

Surgical type	SEMS group (*n* = 33)	ES group (*n* = 50)	*P* value
Left hemicolectomy	10^*∗*^	13^‡^	0.668
Right hemicolectomy	4	17^§^	0.025
Transverse colectomy	2	1	0.712
Sigmoidectomy	17^†^	5	<0.001
Hartmann's procedure	0	14^¶^	0.001

Values are presented as number. ^*∗*^Including one case of multiple organ resection; ^†^including two cases of multiple organ resection; ^‡^including two cases combined with ileostomy; ^§^including one case combined with ileostomy and multiple organ resection and three cases of multiple organ resection; ^¶^including six cases of multiple organ resection.

**Table 3:** Comparison of surgical characteristics and short-term outcomes between the SEMS group and ES group.

Outcomes	SEMS group (*n* = 33)	ES group (*n* = 50)	*P* value^†^
Operative approach			<0.001
Laparoscopic	24 (72.7)	7 (14.0)	
Open	9 (27.3)	43 (86.0)	
Stoma needed	0 (0)	17 (34.0)	<0.001
Resection margin			0.819
*R*_0_	31 (93.9)	45 (90.0)	
*R*_1_	2 (6.1)	5 (10.0)	
Length of surgery (mins)^*∗*^	190.4 (60.6)	209.5 (65.2)	0.182^‡^
Blood loss (ml)^*∗*^	210.6 (199.6)	310.2 (213.3)	0.036^‡^
Postoperative hospital stay (days)^*∗*^	13.3 (10.1)	18.5 (9.5)	0.020^‡^
Overall postoperative morbidity	9 (27.3)^§^	28 (56.0)	0.010
Clavien–Dindo grade			
I	1 (3.0)	5 (10.0)	0.443
II	2 (6.1)	4 (8.0)	0.999
III	1 (3.0)	1 (2.0)	0.999
IV	4 (12.1)	18 (36.0)	0.016
V	0 (0)	4 (8)	0.254

Values are presented as number (%) and ^*∗*^mean (s.d.). ^†^Chi-square or Fisher's exact test, except ^‡^independent *t*-test. ^§^Including one case of stent-related complication. SEMS, self-expanding metal stent; ES, emergency surgery.

**Table 4:** Systematic reviews and meta-analyses on the topic of the SEMS as a bridge to surgery versus emergency surgery for malignant large bowel obstruction.

References	Study type	Study reviewed (number)	Population (SEMS : ES)	Object	Technical/clinical success rate (%)	Findings	
						Significant difference	No significant difference
Tan et al. [4]	Meta-analysis	RCT (4)	234 (116 : 118)	Left colon	70.7/69.0	SEMS: lower overall stoma rate, higher successful primary anastomosis rate	Postoperative mortality, primary anastomosis rate, permanent stoma rate, anastomotic leak rate, surgical site infection rate, 30-day reoperation rate

Ye et al. [16]	Meta-analysis	RCT (3), RS (5)	444 (219 : 225)	Left colon	NA	SEMS: lower overall postoperative morbidity, lower temporary stoma rate	Postoperative mortality, permanent stoma rate, anastomotic leak rate, occurrence of abscesses rate, abdominal complications rate

Zhang et al. [17]	Meta-analysis	RCT (2), RS (6)	601 (232 : 369)	Colon and rectum	87.1/NA	SEMS: lower overall postoperative morbidity, lower overall stoma rate, higher primary anastomosis rate, lower anastomotic leakage rate, lower intensive care rate	Postoperative mortality, permanent stoma rate, overall survival

Cirocchi et al. [18]	Meta-analysis	RCT (3)	197 (97 : 100)	Left colon and rectum	62.9/52.5	SEMS: lower overall stoma rate, higher primary anastomosis rate	Overall postoperative morbidity, postoperative mortality, permanent stoma rate, anastomotic leakage rate, intra-abdominal abscess rate, infections (wound, chest, urinary tract) rate

De Ceglie et al. [19]	Meta-analysis	RCT (5), RS (5), PS (3), CM (1)	876 (405 : 471)	Left colon	96.9/94.2	SEMS: lower overall stoma rate, higher primary anastomosis rate, higher successful primary anastomosis rate, lower infection rate	Postoperative mortality, temporary stoma rate, anastomotic leakage rate, length of hospitalization, overall survival

Zhao et al. [20]	Meta-analysis	RCT (5)	273 (136 : 137)	Left colon	NA	SEMS: lower overall postoperative morbidity, lower overall stoma rate, lower permanent stoma rate, lower surgical site infection rate	Postoperative mortality, primary anastomosis rate, anastomotic leak rate

Huang et al. [21]	Meta-analysis	RCT (7)	382 (195 : 187)	Left colon	76.9^*∗*^/NA	SEMS: lower overall postoperative morbidity, lower permanent stoma rate, higher primary anastomosis rate, lower wound infection rate	Postoperative mortality, anastomotic leakage rate, intra-abdominal infection rate

Amelung et al. [22]	Systematic review	RS (10), PS (4)	2992 (2837 : 155)	Right colon	95.5^*∗*^/89.0^*∗*^	SEMS: lower postoperative mortality, lower major complication rate^†^	Overall postoperative morbidity, minor complication rate^†^

Matsuda et al. [23]	Meta-analysis	RCT (2), RS (7), PS (2)	1136 (432 : 704)	Colon	NA	None	Overall survival, disease-free survival, recurrence rate

Arezzo et al. [24]	Meta-analysis	RCT (8)	497 (251 : 246)	Left colon	NA	SEMS: lower overall postoperative morbidity, lower permanent stoma rate, lower temporary stoma rate, higher primary anastomosis rate	Postoperative mortality

Wang et al. [25]	Meta-analysis	RCT (9)	594 (281 : 313)	Left colon	NA	SEMS: lower postoperative mortality, lower minor complications rate, higher primary anastomosis rate	Anastomotic leakage rate

Amelung et al. [26]	Meta-analysis	RCT (5), RS (12), PS (4)	1919 (938 : 981)	Left colon	NA	SEMS: lower permanent stoma rate	Overall survival, disease-free survival, recurrence rate

Foo et al. [27]	Meta-analysis	RCT (7)	448 (222 : 226)	Left colon	NA	SEMS: lower overall postoperative morbidity, higher recurrence rate	Postoperative mortality, overall survival, disease-free survival

Boland et al. [28]	Systematic review	RCT (7)	408 (201 : 207)	Left colon and rectum	81.1/76.1	NA	NA

Cao et al. [29]	Meta-analysis	RCT (5), RS (16), PS (3)	2580 (1302 : 1278)	Colon and rectum	NA	None	3-year survival, 5-year survival, 3-year disease-free survival, 5-year disease-free survival, local recurrence rate, overall recurrence rate

RCT, randomized clinical trial; RS, retrospective study; PS, prospective study; CM, case-matched; NA, not available. ^*∗*^Value expressed by mean. ^†^Classified by Clavien–Dindo classification of surgical complications: minor morbidity was defined as grade IIIa or lower and major as grade IIIb or higher.

## Data Availability

The data used to support the findings of this study are available from the corresponding author upon request.
